# Multiple gene aberrations and breast cancer: lessons from super-responders

**DOI:** 10.1186/s12885-015-1439-y

**Published:** 2015-05-29

**Authors:** Jennifer J. Wheler, Johnique T. Atkins, Filip Janku, Stacy L. Moulder, Roman Yelensky, Philip J. Stephens, Razelle Kurzrock

**Affiliations:** 1 Department of Investigational Cancer Therapeutics (Phase I Program), The University of Texas MD Anderson Cancer Center, 1400 Holcombe Boulevard, Box 0455, Houston, TX 77030 USA; 2 Department of Breast Medical Oncology, The University of Texas MD Anderson Cancer Center, Houston, TX USA; 3 Foundation Medicine, Cambridge, MA USA; 4 Center for Personalized Cancer Therapy and Division of Hematology and Oncology, University of California at San Diego Moores Cancer Center, La Jolla, CA USA

**Keywords:** Breast cancer, Genomic aberrations, Next generation sequencing

## Abstract

**Background:**

The presence of multiple molecular aberrations in patients with breast cancer may correlate with worse outcomes.

**Case Presentations:**

We performed in-depth molecular analysis of patients with estrogen receptor-positive, HER2-negative, hormone therapy-refractory breast cancer, who achieved partial or complete responses when treated with anastrozole and everolimus. Tumors were analyzed using a targeted next generation sequencing (NGS) assay in a Clinical Laboratory Improvement Amendments laboratory. Genomic libraries were captured for 3,230 exons in 182 cancer-related genes plus 37 introns from 14 genes often rearranged in cancer and sequenced to high coverage. Patients received anastrozole (1 mg PO daily) and everolimus (5 or 10 mg PO daily). Thirty-two patients with breast cancer were treated on study and 5 (16 %) achieved a partial or complete response. Primary breast tissue was available for NGS testing in three of the responders (partial response with progression free survival of 11 and 14 months, respectively; complete response with progression free survival of 9+ months). The following molecular aberrations were observed: PTEN loss by immunohistochemistry, *CCDN1* and *FGFR1* amplifications, and *PRKDC* re-arrangement (NGS) (patient #1); *PIK3CA* and *PIK3R1* mutations, and *CCDN1*, *FGFR1*, *MYC* amplifications (patient #2); *TP53* mutation, *CCNE1*, *IRS2* and *MCL1* amplifications (patient #3). Some (but not all) of these aberrations converge on the PI3K/AKT/mTOR pathway, perhaps accounting for response.

**Conclusions:**

Patients with estrogen receptor-positive breast cancer can achieve significant responses on a combination of anastrozole and everolimus, even in the presence of multiple molecular aberrations. Further study of next generation sequencing-profiled tumors for convergence and resistance pathways is warranted.

**Electronic supplementary material:**

The online version of this article (doi:10.1186/s12885-015-1439-y) contains supplementary material, which is available to authorized users.

## Background

Gene aberrations including, but not limited to, mutations, amplifications and rearrangements drive tumor growth. Aberrations are common in breast cancer in genes such as *HER2* (*ERBB2*), *BRCA*, *PIK3CA*, *TP53*, *GATA3*, *PTEN* and others [1–6]. The presence of multiple gene abnormalities may also serve as an indicator of genetic instability and therefore of poor patient prognosis [7, 8].

Hormone therapy is the treatment of choice for estrogen (ER) and progesterone (PR)-positive breast cancer, but acquired resistance is a significant challenge. The PI3K/AKT/mTOR pathway becomes activated and utilized by cancer cells to bypass the effects of endocrine therapy [9–11]. While investigating the combination of anastrozole (an aromatase inhibitor that blocks estrogen production) and everolimus (an mTOR inhibitor), we noted partial or complete responses (PR or CR) with progression-free survival (PFS) of at least 9 months in five patients with breast cancer of 32 treated [12]. We performed next-generation sequencing (NGS) on three of the responders with available tissue. In depth analysis revealed that, despite their responses, their tumors demonstrated multiple aberrations in genes including *CCND1*, *CCNE1*, *FGFR1*, *MYC*, *IRS2*, *MCL1*, *PIK3CA*, *PIK3R1*, *PRKDC*, and *TP53*. The implications of these diverse aberrations for understanding response and resistance are discussed.

## Case Presentations

At the time of analysis, 32 patients with advanced breast cancer were treated with anastrozole and everolimus, and five attained PR or CR with PFS of at least nine months [12]. In depth analysis was performed using NGS on three responders with available tissue (number of prior therapies = 2, 4, and 7, respectively, in the metastatic setting) (Table 1).

**Table 1:** Clinical Characteristics and Responses of Super-Responders Treated with Anastrozole and Everolimus

Patient No.	1	2	3
Age at Treatment (years)	38	48	44
Date of Diagnosis	August 2009	September 2007	September 2010
Date of Biopsy	August 2009	September 2007	August 2010
Histology	Ductal	Ductal	Ductal
ER Status	90 % Positive	95 % Positive	95 % Positive
PR Status	60 % Positive	80 % Positive	Negative
HER2/neu Status	Negative (FISH)	Negative (FISH)	Negative (IHC)
Prior Treatment in Metastatic Setting	Paclitaxel (3 months) 5-fluorouracil, doxorubicin, cyclophosphamide (1 month) Tamoxifen (3 months) Capacitabine ( 3 months)	Tamoxifen (2 months) Paclitaxel, bevacizumab^a^ Vinorelbine^a^ Fulvestrant (3 months) Ixabepilone^a^ Docetaxel (9 months) Docetaxel, doxorubicin, cyclophosphamide (3 months)	Tamoxifen, zoledronic acid (6 months) Letrozole (4 months)
Prior Treatment with Aromatase Inhibitor in Metastatic Setting (Duration)	No	No	Yes (4 months)
Molecular alterations (Please reference Additional file 1: Table S1)			
Progression Free Survival (months)	11	14	9+
Best Response (%)	−56 %	−38 %	−100 %

^a^Unknown duration of treatment

*FISH* Fluorescent in-situ hybridization, *IHC* Immunohistochemistry

*Patient #1* is a 38-year old woman with ER-positive (90 %), PR-positive (60 %), HER2-negative, invasive ductal carcinoma diagnosed in August 2009. The patient was referred to the Clinical Center for Targeted Therapy at MD Anderson Cancer Center in February 2011. Previous therapies in the metastatic setting included: (1) paclitaxel; (2) 5-fluorouracil, doxorubicin, cyclophosphamide; (3) tamoxifen; and (4) capecitabine. Immunohistochemistry revealed complete nuclear PTEN loss. NGS of left breast tumor tissue dated August 2009 revealed amplifications in *CCDN1* and *FGFR1* in addition to a rearrangement in *PRKDC*.

**Fig. 1:**
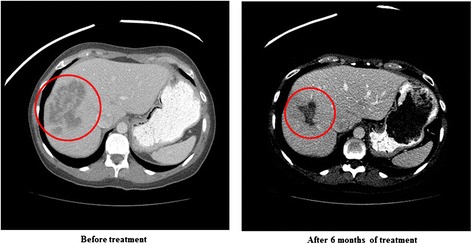
Patient #1 CAT scans of the abdomen show a partial response (56 % decrease in hepatic disease) after 6 months of treatment. Patient received treatment for 11 months before progressing

The patient was treated with anastrozole 1 mg PO daily and everolimus 5 mg PO daily beginning March 2011. She attained a PR (56 % decrease in liver metastases) (Fig. 1) and remained on study for 11 months.

*Patient #2* is a 48-year old woman with ER-positive (95 %), PR-positive (80 %), HER2- negative, invasive ductal carcinoma diagnosed in September 2007. At that time, metastatic disease was found in the pleura and bones. The patient was referred to our clinic in March 2011. Previous therapies in the metastatic setting included: (1) tamoxifen; (2) paclitaxel with bevacizumab; (3) vinorelbine; (4) fulvestrant; (5) ixabepilone; (6) docetaxel; and (7) docetaxel, doxorubicin and cyclophosphamide. Immunohistochemistry showed PTEN to be present. NGS of malignant tissue from the left breast dated September 2007 revealed mutations in *PIK3CA* and *PIK3R1* in addition to amplifications in *CCDN1*, *FGFR1* and *MYC*.

The patient was treated with anastrozole 1 mg PO daily and everolimus 5 mg PO daily starting March 2011. She achieved a PR (38 % decrease in measureable disease). After 14 months on study, she showed signs of progression. At that time, a third agent, fulvestrant (an estrogen receptor antagonist) was added as per protocol for triple combination therapy. Five months later, she continues on this triple-agent treatment.

*Patient #3* is a 44-year old woman with ER-positive (>95 %), PR-negative, HER2-negative, invasive ductal carcinoma diagnosed in September 2010. The patient was referred to our clinic in October 2011. Previous therapies in the metastatic setting included: (1) tamoxifen with zoledronic acid and (2) letrozole (progression free survival = 4 months). Immunohistochemistry showed intact PTEN. NGS of tissue from the primary left breast tumor at the time of diagnosis revealed a mutation in *TP53* and amplifications in *CCNE1*, *IRS2* and *MCL1*.

**Fig. 2:**
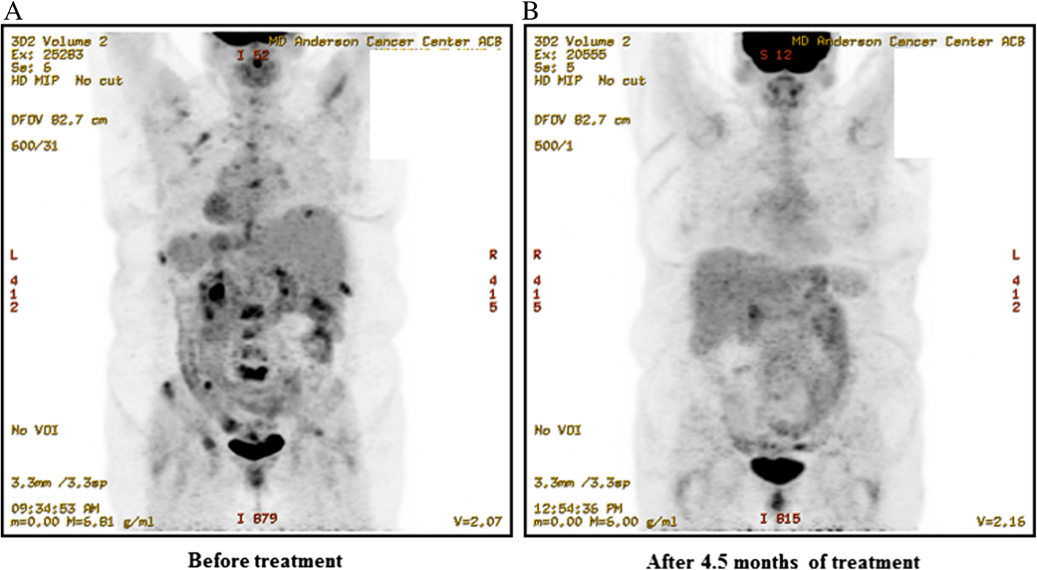
Patient #3 PET scans show metastatic bone disease that attained a complete metabolic response after 4.5 months of treatment. Patient remained on treatment after 9 months at the time of this analysis

The patient was treated with anastrozole 1 mg PO daily and everolimus 10 mg PO daily. At the time of treatment her metastatic disease was only in bone. She achieved a CR noted on PET/CT (Fig. 2) (time on study = 9+ months).

We present an in-depth analysis of three patients with advanced, refractory breast cancer who attained remarkable responses despite the fact that their tumors harbored multiple gene alterations (Additional file 1: Table S1). Of interest, in each case, despite the complexity of aberrations revealed by NGS, many of the molecular abnormalities can be shown to converge, at least in part, on the PI3K/AKT/mTOR axis, perhaps accounting for the responses. On the other hand, some of the genes involved also modulate other pathways, and this fact may explain why two of the patients attained only partial responses, and eventually relapsed.

In patient #1, who had ER/PR-positive disease, abnormalities included: *PTEN* loss; *CCDN1* and *FGFR1* amplifications and a re-arrangement in *PRKDC*. Yet, she achieved a durable PR on anastrozole and everolimus. PTEN loss is known to activate the PI3K pathway [13], which might easily explain the response to the mTOR inhibitor everolimus. Of interest, however, the other aberrant genes detected in this patient’s tumor might also affect this pathway. For instance, *CCND1*, also known as *BCL1*, is down regulated by mTOR inhibition [14, 15]. *FGFR1* encodes a tyrosine kinase receptor belonging to fibroblast growth factor receptor (FGFR) family; this gene is amplified in 9 to 15 % of breast cancers [16–18]. Activation of FGFR induces PI3K and AKT pathway activity through recruitment and tyrosine phosphorylation of the docking protein Gab [19]. Finally, *PRKDC* was rearranged in patient #1. This gene is a member of the PI3K family and encodes the catalytic subunit of the DNA-dependent protein kinase (DNA-PK). It functions with the Ku70/Ku80 heterodimer protein in DNA double strand break repair and recombination [20]. Overall, though there were several different aberrations in this patient, each has some impact on PI3K/AKT/mTOR signaling. Yet, some of the aberrations (e.g., *FGFR1*) also activate other signaling pathways such as MAP kinase [21], which might explain why complete response was not attained and/or the eventual progression that developed after 11 months.

In patient #2, who also achieved a durable PR (14 months) on anastrozole and everolimus, multiple aberrations were seen as well. NGS of malignant tissue revealed mutations in *PIK3CA* and *PIK3R1*, in addition to amplifications in *CCDN1*, *FGFR1* and *MYC*. Responses of patients with *PIK3CA*-mutant breast, gynecologic and other tumors to mTOR inhibitors has been previously reported [3, 22–26]. The crosstalk between *CCND1* and *FGFR1* (both amplified in this patient) and the PI3K/AKT/mTOR pathway has been discussed above. *MYC* encodes a transcription factor with multiple functions,[27] including interaction with the PI3K/AKT/mTOR axis as evidenced by the observation that MYC-induced proliferation and transformation require AKT-mediated phosphorylation of FoxO proteins [28]. Of interest, both this patient and patient #1 harbored *FGFR1* aberrations and, as mentioned, *FGFR1* can activate both PI3K and MAPK signals, hence potentially explaining both initial response to everolimus (via PI3K activation) and eventual resistance (via MAPK). *MYC* might also play a role in limiting the response of this patient to less than a CR, since *MYC* regulates expression of a broad array of genes via its functions as a transcription factor, transcriptional repressor, and regulator of global chromatin structure by means of recruitment of histone acetyltransferases [29].

Patient #3, who attained a CR on therapy, demonstrated a mutation in *TP53* and amplifications in *CCNE1*, *IRS2* and *MCL1. TP53* is a regulator of *PTEN*, which is in itself a negative regulatory of *PIK3CA* [30]. *CCNE1* degradation is regulated by *GSK*-*3*β, which is directly phosphorylated by *AKT* [31, 32]. Insulin receptor substrates (IRS) serve as downstream messengers from activated cell surface receptors to numerous signaling pathway cascades including PI3K [33]. Finally, mTORC1 promotes survival in part through translational control of *MCL*-*1* [34]. Previous studies have demonstrated up-regulation of the PI3K/AKT/mTOR pathway as a mechanism of resistance to hormone therapy [35–40], and clinical trials combining hormone therapy with mTOR inhibitors have shown promise in breast and endometrial cancers [41–43]. Further, the combination of exemestane (an aromatase inhibitor) and everolimus was FDA approved for metastatic breast cancer. Other possibilities for the response in this patient may be the effect of anastrazole or the development of new molecular alterations since the primary diagnosis as molecular profiling was done on primary breast tissue.

In our study of anastrozole and everolimus in hormone receptor-positive breast cancers we observed salutary activity in patients with multiple gene aberrations. Patient #1 and Patient #2 demonstrated direct alterations in the PI3K/AKT/mTOR pathway (PTEN loss and *PIK3CA* mutation, respectively) in addition to multiple gene amplifications and additional rearrangements or mutations. It is therefore plausible that these patients responded because their tumors maintained dependence on or addiction to the PI3K/AKT/mTOR axis despite the presence of additional gene alterations. Recently, Hortobagyi et al. [25] reported that patients treated on the Phase 2 BOLERO-2 study had a greater treatment effect if they had no or only 1 genetic alteration in *PI3K* or *FGFR* pathways or *CCND1*. Some of the additional molecular alterations demonstrated by NGS in our patients may however also modulate the PI3K/AKT/mTOR axis, as noted above, explaining our findings. Systems biology approaches that map convergence pathways may therefore enhance treatment for patients [5, 6, 44, 45]. Alternatively, an eventual relapse (and in patients #1 and #2, partial, rather than complete, response) could be the result of the actions of the additional molecular alterations. Of interest, at the time of progression, fulvestrant, an estrogen receptor antagonist, was added to anastrozole and everolimus in patient #2, with a 5+ month disease stabilization. This strategy (adding an agent rather than changing regimens) is consistent with observations in patients with HER2-positive metastatic breast cancer that continuation of trastuzumab beyond progression can be beneficial [46] as well as our observations on resistance and retreatment [47]. The mechanism is unclear but could relate to residual sensitive tumor cells that co-exist with the emerging resistant clone.

## Methods

### Patients

As part of a dose escalation study of the aromatase inhibitor anastrozole and the mTOR inhibitor everolimus (NCT01197170), we performed NGS on patients who had attained PR or CR (and had a PFS of at least nine months) and available tissue. NGS testing was not a part of standard care, but was done retrospectively after responses were seen. Research involving human subjects (including human material or human data) that is reported in this study was conducted in accordance with the guidelines of the MD Anderson Internal Review Board, and patients signed informed consent for all experimental therapeutic interventions and for the deposit of NGS results.

### Evaluation of HER2/neu amplification, estrogen and progesterone receptor status

Under CLIA conditions, immunohistochemistry was used to measure of HER2/*neu*, estrogen and progesterone receptors. Estrogen and progesterone receptors were assessed using antibody 6 F11 (Novocastra Laboratories, Ltd., Newcastle Upon Tyne, UK). Alternatively, fluorescence in situ hybridization (FISH) was used to measure the copy number of HER2/*neu*.

### Next-generation sequencing

Molecular analysis using NGS was performed on archival formalin-fixed, paraffin-embedded tissue. Genomic libraries were captured for 3230 exons in 182 cancer-related genes plus 37 introns from 14 genes often rearranged in cancer and sequenced to average median depth of 734× with 99 % of bases covered >100× (Foundation Medicine, Cambridge, MA, USA).

### Evaluation of PTEN expression

PTEN expression was assessed using a Dako antibody (Carpinteria, CA, USA) as previously published [49, 50].

### Evaluation of response

Responses were assessed after three cycles (about 12 weeks) or earlier at the discretion of the treating physician. All radiological tests were assessed by an MD Anderson radiologist. In addition, results were reviewed in a departmental tumor measurement clinic and by the attending physician. RECIST criteria were used for progressive disease (PD), stable disease (SD), partial and complete responses (PR and CR).

## Conclusions

In each of these “super-responders,” tumor tissue that was derived from diagnosis was analyzed by NGS. It is conceivable that the number of aberrations increased by the time the patient was treated in our clinic, which was between one and 3.5 years after diagnosis, especially since each of these patients had received multiple intervening therapies, and now had advanced metastatic disease. Despite these limitations, NGS on tissue from diagnosis was informative, and revealed abnormalities that were actionable, similar to an earlier report by Kalinsky et al. [48]. Optimizing future studies probably requires fresh biopsies at the time of each relapse. However, in their absence, tumor tissue derived from an earlier point in time still yields useful information. Our observations suggest that NGS can help unravel the mechanisms of response and resistance, and warrants additional investigation as a clinical tool to optimize treatment.

### Consent

Ethical approval of this study was obtained from the MD Anderson Internal Review Board. Each patient willingly provided written informed consent for all treatment related activities, including the publication of individual outcomes, accompanying images and the deposit of NGS data into an institutional database.

## Additional file

Additional file 1: Table S1. Molecular Alterations of Super-Responders Treated with Anastrozole and Everolimus.
