# Supplementary material for: Multiple gene aberrations and breast cancer: lessons from super-responders
Source: BMC Cancer. 2015 May 29;15:442. doi: 10.1186/s12885-015-1439-y (PMC4446801; doi:10.1186/s12885-015-1439-y)
Supplement: Additional file 1: Table S1. — Molecular Alterations of Super-Responders Treated with Anastrozole and Everolimus. [file 12885_2015_1439_MOESM1_ESM.docx]

**Additional file 1: Table S1**

| **Patient No.** | **1** | **2** | **3** |
| --- | --- | --- | --- |
| **Molecular Alterations** | PTEN loss (100% nuclear, 70% cytoplasmic),  *CCND1* amplification,  *FGFR1* amplification,  *PRKDC* re-arrangement | *PIK3CA* mutation (H1047R),  *PIK3R1* mutation (G376R),  *CCND1* amplification,  *FGFR1* amplification,  *MYC* amplification | *TP53* mutation (I195T),  *CCNE1* amplification,  *IRS2* amplification,  *MCL1* amplification |
